# A comprehensive method for quantifying human cytomegalovirus plaque assays

**DOI:** 10.3389/fcimb.2026.1818664

**Published:** 2026-04-24

**Authors:** Laurel E. Kelnhofer-Millevolte, Daniel H. Nguyen, Lea S. Wilson, Daphne C. Avgousti

**Affiliations:** 1 Molecular and Cellular Biology, Graduate Program, University of Washington and Fred Hutchinson Cancer Center, Seattle, WA, United States; 2 Medical Scientist Training Program, University of Washington, Seattle, WA, United States; 3 Department of Molecular and Cellular Pharmacology at the University of Miami Miller School of Medicine, Miami, FL, United States; 4 Cancer Epigenetics Group at the Sylvester Comprehensive Cancer Center at the University of, Miami, FL, United States; 5 Human Biology Division, Fred Hutchinson Cancer Center, Seattle, WA, United States

**Keywords:** herpesvirus, human cytomegalovirus (CMV), imaging, infection, plaque assay

## Abstract

Human cytomegalovirus (hCMV) is an important human pathogen accounting for significant morbidity in immunocompromised individuals from neonates to cancer patients. To effectively produce progeny and establish a new infection, hCMV produces dozens of proteins and manipulates the expression of thousands of host genes. When studying the effect of these viral or host genes on the production of new infective progeny, the plaque assay is the gold standard method employed. This assay is carried out by incubating serial dilutions of the supernatant to be investigated on a monolayer of permissive cells. The experimental setup includes covering the monolayer with an overlay, thereby allowing the virus to spread only through cell-to-cell contact. Subsequently, the areas of cell death, or plaques, are quantified by fixing and staining with crystal violet. Here we take advantage of the fluorescent nature of crystal violet and image hCMV plaques in a variety of modalities. Following the acquisition of these images, we developed a broadly applicable pipeline to use open access software ImageJ to quantify the number and size of the hCMV plaques. The use of this method can provide a standardized way to objectively count and quantify the infectious progeny produced, allowing researchers to better understand hCMV.

## 1 Introduction

Herpesviruses are large DNA viruses that encode a vast array of proteins involved in the production of infectious viral progeny. Many of the human herpesviruses are known for their minor effects, such as sores and cold-like symptoms. In contrast, herpesviruses can also cause severe diseases in neonates (Samies and James, 2020) and are a major source of morbidity following transplantation (Azevedo et al., 2015; Kotton et al., 2018), while gammaherpesviruses are oncogenic (Pei et al., 2020). Nonhuman herpesviruses can have devastating agricultural and economic impacts in cattle (Sayers, 2017), pigs (Liu et al., 2022), poultry (Davidson, 2020), fish (Klafack et al., 2022), and even mollusks (Renault et al., 2014). The extensive human health and economic burdens of herpesviruses highlight the need for continued research on the basic biology of herpesviruses.

In a research setting, human cytomegalovirus (hCMV) is considered a slow-growing herpesvirus (Alwine, 2012; Knipe and Howley, 2013; Turner and Mathias, 2022; Dotto-Maurel et al., 2025) that poses many unique challenges to study. Many laboratory strains of hCMV only replicate in specific cell types such as fibroblast cells (Harmenberg and Brytting, 1999). The virus itself is defined by a prolonged infection cycle, with replication in cell culture peaking between 72 and 120 h post-infection *in vitro*, depending on the cell type and virus strain (Tandon and Mocarski, 2012; Knipe and Howley, 2013; Mocarski, 2024). This delayed infection cycle brings increased potential for cell death unrelated to infection and requires monitoring over several days.

In investigating the roles of various viral and host proteins in viral replication and progeny production, plaque assays are the gold standard (Boeckh and Boivin, 1998; Knipe and Howley, 2013; Akhtar et al., 2019). Plaque assays rely on infection and subsequent death of cells in a monolayer. To accurately measure infectious progeny production as plaque-forming units per volume of sample, serial dilutions are carried out in triplicate. To ensure that plaques form in localized areas of the monolayer, an overlay is usually employed to prevent infections through the media and restrict them to cell-to-cell contact. Several cells must be infected and die for a plaque to form. Therefore, these assays rely on multiple rounds of viral replication, prolonging hCMV experiments even further. Many people engaging in hCMV research employ assays that use GFP reporters or other proxies to measure infectivity (Sampaio et al., 2005, 2017). While these assays have been extremely useful to the field, they are inherently limited to the output being measured—for example, a GFP fusion may affect viral protein function, or using viruses that express GFP on ubiquitous promoters provide information only measuring the viral entry or expression of the reporter gene rather than the full infection. Similarly, assays that use staining of Immediate Early Proteins (IE1/2) (Gardner et al., 2013) are limited to the expression of a single protein representing only the early stages of infection. Furthermore, because of the distinct morphology of fibroblast cells and the slow nature of hCMV plaques (Das et al., 2014; Kelnhofer-Millevolte et al., 2025), it can be challenging to identify a single plaque formed. Therefore, we have developed a new combination method of imaging plaques together with digital quantification of stained cells to remove some of the subjectivity of plaque identification.

Here we present a protocol for hCMV plaque assays that improves the standard assay by layering on ImageJ quantification of plaques. This allows for the standardization of data on plaque growth to be efficiently calculated, maximizing information gleaned while minimizing variability from plaque assays. Though this paper focuses on hCMV plaques, we anticipate that the digital quantification method can be adapted for other species and cell types or readily applied to the quantification of plaques from other viruses.

## 2 Materials

### 2.1 Consumable materials

(Listed as company; catalog number).

**Figure 1:**
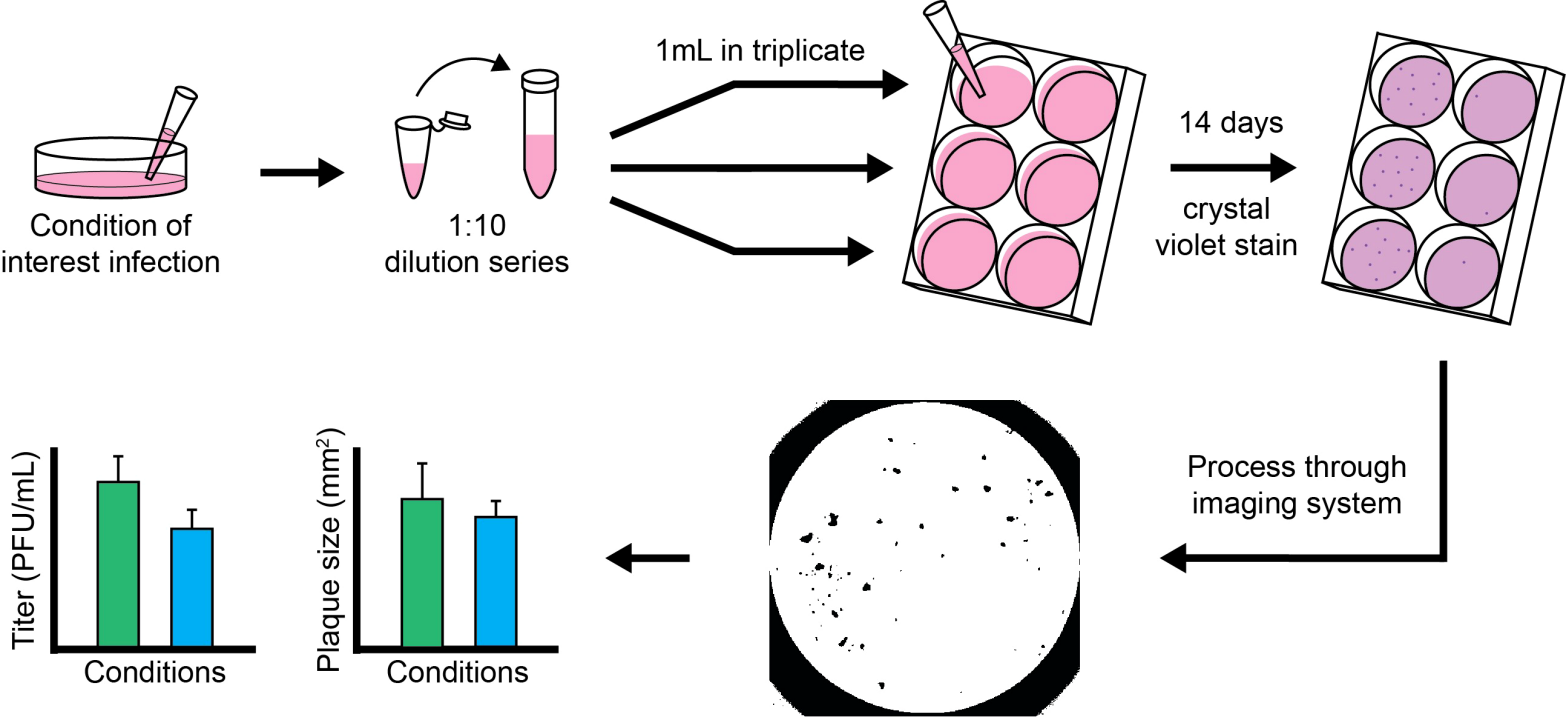
Schematic of hCMV experimental workflow. Key steps include preparation of plaque assays, imaging, image processing, and data analysis.

Note: Many similar products exist from other companies and can be used interchangeably with the products listed below. Importantly, if supernatant from experimental conditions will be frozen prior to running plaques assays, it is critical to ensure that the microcentrifuge tubes are rated to -100°C or that cryovials are used for freezing. Human foreskin fibroblasts with hTERT (HFF-T)-immortalized cells can be more sensitive than other cell lines to variations in manufacturing of tissue-culture rated plastics, which can lead to the poor formation of a cell monolayer. If plates are not 100% confluent after ~36 h of plating, consider using the catalog numbers listed below. Once the cells are ready, proceed with the experimental setup as listed in **Figure 1**.

- 70% ethanol.
- Lysol—diluted 15 mL per 1 L water (Lysol; 74392).
- Methyl cellulose (Sigma-Aldrich; M0387).
- Anhydrous crystal violet (Sigma-Aldrich; C6158).
- 50-mL centrifuge tubes (Corning; 430828).
- 15-mL centrifuge tubes (Corning; 352096).
- Cryovials, 2-mL volume (Corning; 430659).
- 2-mL microcentrifuge tubes (Eppendorf-Fisher Scientific; 022364111).
- Six-well TC plates (Fisher Scientific; 130184).
- DPBS (Fisher Scientific; 14190250).
- Trypsin (Fisher Scientific; 25200114).
- Gibco high-glucose DMEM (Thermo Fisher Scientific; 11965118).
- Gibco penicillin–streptomycin 10,000 U/mL (Fisher Scientific; 15140163).
- Heat-inactivated fetal bovine serum (FBS) (Sigma-Aldrich; F0926).
- 1-L corning bottles (Millipore Sigma; CLS13951L).
- Liquid nitrogen.

### 2.2 Equipment

Note: Equivalent equipment can be used and provide comparable settings to the parameters listed below:

- Tabletop centrifuge (Fisher Scientific; Sorvall Legend XTR).
- Vortex mixer (Fisher Scientific; 88880017).
- Dissecting scope (VWR; 470314-882).
- Low-speed orbital shaker (Corning LSE, CLS6781FP-1EA).
- Zeiss LSM 980 confocal microscope OR.
- Bio-Rad ChemiDoc™ MP Imager (Bio-Rad; 1200315) OR.
- Smartphone.

### 2.3 Software

Note: A link to a basic ImageJ macro is included here. The macro is annotated using “//” to describe parameters and how to adjust the said parameters for acquired images of plaque assays. Section 3.7 includes more detailed explanations of the image analysis, and should a user wish to alter the parameters, it may be preferable to record a new macro without using the link below. The instructions provided here are listed for use with ImageJ software version 2.1.0 java 1.8.0_172 and Zeiss software ZEN version 3.11.105.06000. Adjustments may be needed for newer versions released after this protocol was developed.

- ImageJ software available for free at https://imagej.net/ij/.
- https://zenodo.org/records/18772333.

### 2.4 Cells and viruses

This protocol was optimized in hTERT-immortalized human foreskin fibroblast (HFF-T) cells (Phelan, 2006; Nadalutti and Wilson, 2020) using the Towne strain of hCMV (Kemble et al., 1996) based on existing methods recommended from several lab experts in hCMV biology (Vieira et al., 1998; Lee et al., 2015; Liu et al., 2017; Britt, 2018; Albright et al., 2021; Zarrella et al., 2023; Mahmud et al., 2024; Mokry and Purdy, 2024). hTERT-immortalized HFFs are derived from various donors, and thus there is a likely variation between cell lines. The timing of cell growth and virus incubation post-infection should be considered as a starting point for different cells or viral strains, though optimization may be required on the timing of infections if other cell lines or viral strains are used. Most hCMV strains replicate best in fibroblast cells. However, some viral strains have been modified to allow for infection in other cell lines. For hCMV strain and cell line compatibility, see **Table 1**.

**Table 1:** Viruses and approximate time frames for infections.

Strain	Average time to plaque fixation	Cell type	Cell/media suggestion
*Towne*	2 weeks/14 days	Fibroblasts	HFF-T cells, DMEM
*TB40/E* (Vo et al., 2020)	3 weeks/21days	Endothelial cells, fibroblasts, epithelial cells	HFF-T cells, DMEM
*BADrUL131* (Wang and Shenk, 2005)	3 weeks/21 days	Epithelial cells. endothelial cells, neuronal cells, fibroblasts	RPE-1 cells, DMEM:F-12K
*AD169* (Yu et al., 2002)	3 weeks/21 days	Fibroblasts	HFF-T cells, DMEM

HCMV strain, recommended cell type for experiments using corresponding strain, average time to plaque fixation using the recommended protocol presented in section 3.1-3.5, and recommended media for sections 3.1-3.5 based on cell type.

Prior to the experimental setup, HFF-T cells should be cultured in 10% FBS (see the recipe in Section 6.5) in 15-cm TC-grade plate, grown at 37°C with 5% CO_2_, and routinely tested for mycoplasma contamination. Cell line identity should also be verified using SNP assay or genetic sequencing. Viral stocks used for experimentation should be harvested as established for the strain. Information on the *Towne* strain can be found in other publications (Kemble et al., 1996).

## 3 Protocol

The following sections include detailed steps for both setting up hCMV plaque assays and subsequent imaging and analysis of fixed plaques. Many labs have established protocols that reliably generate reproducible plaques. Minor differences are unlikely to impact final data collection. Those familiar with hCMV plaque assays may proceed directly to step 3.6 for imaging and processing.

### 3.1 Harvesting experimental hCMV sample

Plate experiment as desired, for example, siRNA or inhibitor treatment as in Kelnhofer-Millevolte et al. (2025) and Shin and Kim (2026).

For experimental infections, dilute the virus to the desired MOI in serum-free media. MOI is based on experimental question; for our experiments, we use an MOI of 1. Incubate the viral inoculum with cells for 1 h, with shaking every 10 min to ensure infection. If the lab has a rocker or similar machine in cell incubators, it can be used here. If not, periodic rocking of plates by hand is sufficient. After this 1 h, replace the viral inoculum with 4%-FBS-containing media (see the recipe in Section 6.4) as per **Table 2** to allow for an adequate volume of supernatant at harvest. For the *Towne* strain, we recommend supernatant harvest between 4 and 6 days post-infection.

**Table 2:** Recommended inoculum volumes for infection.

Plate/dish size	Inoculum volume	Volume of media to add after infection
15-cm plate	3 mL	17 mL
10-cm plate	1 mL	9 mL
Six-well plate	500 µL–1 mL	1.5–1 mL/well

Recommended volume of media to be used in section 3.1 based on experiment size.

1. Transfer the supernatant volume into a 15-mL centrifuge tube. Note: For larger experiments, the supernatant can be collected into multiple 15-mL centrifuge tubes or 50-mL centrifuge tubes as needed.
2. Centrifuge at 3,000*g* for 2 min to remove cellular debris.
3. Aliquot into 1-mL volumes using cryovials or microcentrifuge tubes.
4. Flash-freeze the cryovials in liquid nitrogen (or a slurry of dry ice with 100% ethanol) before storing the aliquots in -80°C, or proceed directly to dilution (step 3.3).

Note: Dry ice – ethanol slurry takes longer to freeze harvested pellets. Additionally, if choosing this mixture, it will need to be made ahead of time to ensure that the samples are preserved properly.

### 3.2 Preparation of cells for plaque assays

We recommend using six-well plates rather than 12-well plates for hCMV plaque assays because they result in less variation between wells. Furthermore, we recommend at least three serial dilutions per condition to be tested in triplicate as described below. We have listed an example of an infection below, with the *Towne* strain on HFF-T cells which require DMEM. Adjust the cell line and media according to **Table 1** depending on the hCMV strain.

Day 0:

1. Aspirate media from HFF-T cells. Wash two times with 10 mL of 1× PBS, aspirating after each wash. Add 3 mL trypsin to the plate. Incubate the plate at 37°C for 5–10 min.
2. Use 9 mL of DMEM with 10% FBS and gently triturate HFF-T cells to lift them from the plate. Transfer the cells and media to a 15-mL centrifuge tube. Centrifuge at 500*g* for 5 min.
3. Aspirate supernatant; be careful not to disturb the cell pellet. Resuspend the cell pellet in 10 mL DMEM with 10% FBS. Count the cells using a hemocytometer for fibroblasts as automated cell counters are less reliable with large cells such as fibroblasts.
4. Dilute the HFF-T cells to ~210,000 cells/2 mL using DMEM with 10% FBS. Ensure that the cells are well distributed in the media. Plate 2 mL of cell/media mixture per well. Shake the plate along the X and Y axes to distribute the cells uniformly.
5. Incubate the plates overnight at 37°C.

### 3.3 Dilution of samples for plaque assays

Note: Prior to preparing the viral dilutions, check that the cells plated in step 3.2 are 100% confluent. If the cells are not confluent, wait another day for the cells to grow before starting the plaque assay. Additionally, even a single freeze–thaw cycle of hCMV can reduce the titers of the virus by over 10-fold and will decrease the reproducibility of the assay. It is critical to not thaw the virus until the cells are ready for infection. The recommended dilutions for the *Towne* strain are 10^−2^ to 10^−4^ for high-MOI initial experiments and undiluted to 10^−2^ for low-MOI initial experiments. Common high-MOI experiments for hCMV range from 1 to 3, and for low MOI, this ranges from 0.1 to 0.

**Figure 2:**
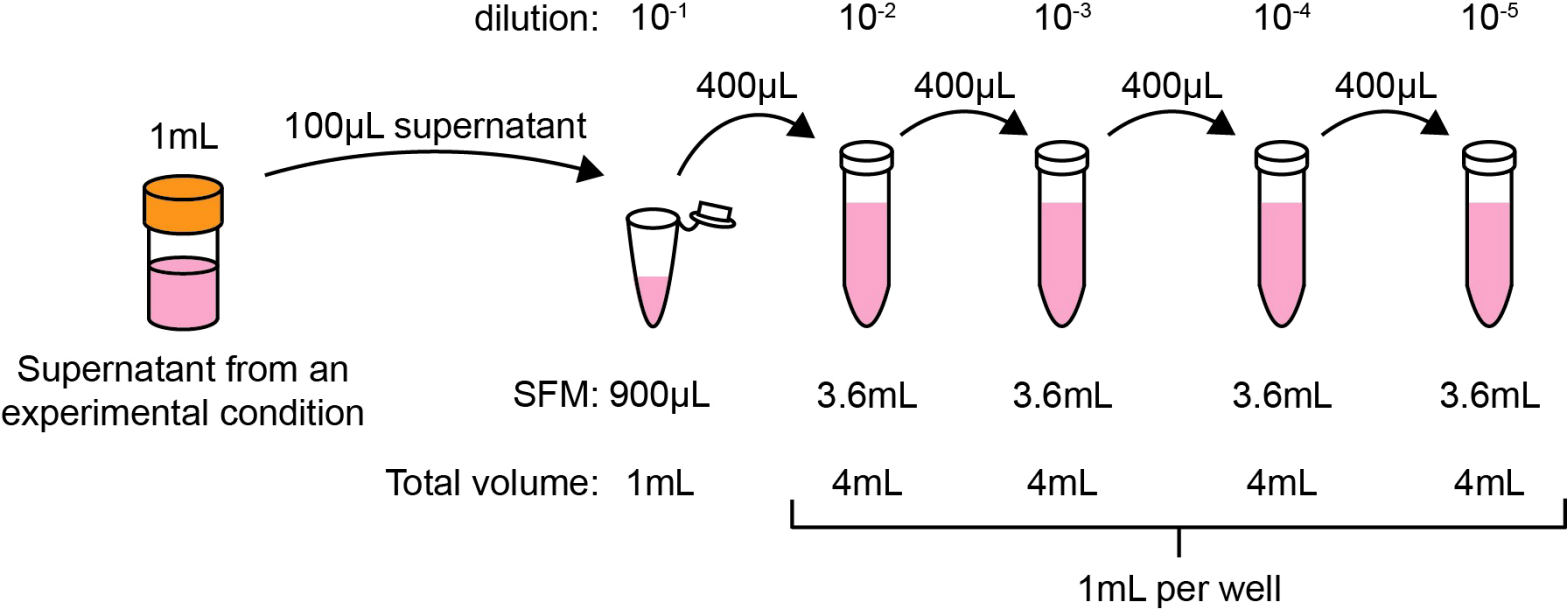
Schematic of standard dilution series for hCMV plaque assays as described in Section 3.3.

Day 1:

1. Thaw the viral aliquot with an unknown titer in 37°C water bath. Note: If conducting a plaque assay for a viral stock, we recommend a brief water bath sonication (1 min on, 1 min off to rest, 1 min on) of the samples to ensure the release of any virus that is still cell-associated.
2. Label the microcentrifuge and 15-mL centrifuge tubes with dilution and conditions. Add 3.6 mL of serum-free DMEM (see the recipe in Section 6.3) to each tube. For 10^−1^ dilution, start with 900 µL of serum-free DMEM.
3. Vortex the viral aliquot for 30 s. Gently pipet up and down at least five times prior to the transfer of the inoculum. This is important to coat the pipet tip and ensure the accurate transfer of the virus.
4. Transfer 100 µL of the supernatant from the experimental condition into the 10^−1^ vial. Discard the pipet tip.
5. Vortex the dilution for 30 s. Using a fresh pipet tip, triturate three times.
6. Transfer 400 µL into the next dilution tube. Discard the pipet tip.
7. Repeat steps 5 and 6 until the end of the desired dilution series (**Figure 2**).

### 3.4 Infecting cells for plaque assays

(Continued from Section 3.3).

8) Remove the six-well plates from the incubator and aspirate media. Wash all wells with 1 mL of PBS each. Aspirate PBS. Take care not to disturb the monolayer.

9) Vortex each dilution sample for 10 s. Immediately after vortexing the sample, add 1 mL of the inoculum to each well. Dispense each dilution in three separate wells.

10) Incubate the plates for 1 h. Agitate the plates every 10 min to encourage even viral coating of the entire monolayer.

11) Incubate the plates with inoculum on the cells overnight.

Day 2:

1. Prepare the overlay in 50-mL centrifuge tube(s). Each well requires 2 mL of overlay. Pour ~15 mL 2% methylcellulose (see the recipe in Section 6.1) into the centrifuge tube first before gently pipetting 15 mL DMEM with 4% FBS on top.
2. Warm the overlay in the 37°C water bath for a minimum of 30 min. Gently triturate the overlay several times, taking care not to shake and add bubbles.
3. Remove the infected plates from the incubator. Aspirate inoculum, taking care to use a fresh tip for each dilution.
4. Wash each well with 1 mL of 1× PBS; aspirate the PBS.
5. Gently pipet 2 mL of methylcellulose-based overlay into each well. Pipet slowly to limit the formation of bubbles. Supporting the tip of the pipet on the edge of the well also minimizes the disruption of the cell monolayer as overlay is added to the well. Avoid large bubbles.
6. Halfway through the extended incubation period, gently layer an additional 1 mL of warmed DMEM with 4% FBS.

### 3.5 Fixing plaque assays

Note: Check the size of plaques forming on the monolayer daily on days 10–14. When holding the plate up to a light source, the plaques will be visible as white spots on the plate. If they are visible by eye, they are ready to fix. For the *Towne* strain, this occurs approximately at day 14. For the average time to fix for other strains, refer to **Table 1**. Once the timing has been optimized, keep consistent among all conditions for a most accurate plaque size quantification.

Day 14:

1. Aspirate approximately half of the overlay. Do this by tilting the plates to ~60° while aspirating half of the side of the well. Note: This is recommended to reduce the disruption of the cell monolayer. Other labs have had success using less viscous material for overlay such as Avicel (Matrosovich et al., 2006), although we recommend using a clear overlay for the hCMV plaque assays, such as agarose or methylcellulose, to allow visualizing the cells during the assay. It is important to be able to view the cells over the long incubation period to monitor plaque formation.
2. Gently add 1 mL of 1× PBS to each well, layering on top of the remaining overlay.
3. Aspirate half of the PBS/inoculum using the same method as in step 1.
4. Repeat steps 2 and 3 two more times. The overlay should be clear by the third wash. If not, repeat steps 2 and 3 two more times.
5. Add 0.5 mL of 1% crystal violet (see the recipe in Section 6.2) to each well.
6. Gently shake the plates to ensure that the crystal violet spreads evenly and place on a low-speed orbital shaker at RT for 15 mins.
7. Remove most of the crystal violet by flipping the six-well plate over a funnel into a waste tank. Tap the plate against the opening of the funnel until the crystal violet splatter no longer shows. Note: Ensure that the crystal violet is handled according to institutional safety protocols. This may require the removal of crystal violet and washing with deionized (DI) water using a pipet and moving the liquid directly into a disposal canister.
8. Wash the wells repeatedly by dunking the plate right side up in a tub of DI water to minimize damage to the fixed monolayer. Wash the wells until the contrast of the plaques to the cell monolayer is easily visible.
9. Place the six-well plates on a bench pad upside down and at an angle to dry; this can be achieved by leaning the plate against the lid. Allow the plaques to dry at least overnight prior to imaging.

### 3.6 Imaging plaques

Note: The imaging for this protocol was originally optimized in our previous paper (Kelnhofer-Millevolte et al., 2025) using Molecular Devices ImageXpress Micro high-content imaging system. Here we present multiple additional modalities to image the fixed plaque assays. Our goal is to make the protocol more accessible by providing many options for imaging based on equipment available in the lab. The advantages and disadvantages of each imaging modality are presented in the “Discussion”. Wells imaged for analysis should have 10–100 plaques and have minimal plaques touching other plaques. If no wells meet these criteria (see “Discussion” for troubleshooting). Wells only need to be imaged using one method. After images are gathered, proceed to Section 3.7 for image analysis. If the volume of plaques is not desired, the measurement ruler placement can be omitted.

Imaging option 1—Confocal microscope:

1. Place the fixed plates from Section 3.5 with the plastic bottoms of wells facing the microscope objective without lid in the plate holder area. Here we use a Zeiss LSM 980 confocal microscope with a sample finder, but other comparable confocal microscopes can be used.
2. Set the imaging setting to acquire fluorescence images with a Cy5 (647-nm excitation) filter set. Collect images using a 10× objective.
3. Open the acquisition tab and select “AI Sample Finder”. Select the “Center Position” and “Find Sample buttons”. The AI Sample Finder will automatically identify the individual wells as regions of interest (ROIs) in the multiwell plate and generate an overview image with the wells highlighted.
4. Select the “tiles” option from the main menu. In the tiles menu that opens, select “Show viewer”. Select “Setup from sample carrier” option for the tile type.
5. Select “Fill Factor” located in the top menu. Set the fill factor to 100%, and then select 5) all wells from the carrier menu and select the + sign.
6. Set the tile size to 850.1 μm × 709.3 μm for a standard six-well plate. Activate tiles at 10% overlap.
7. Add support points throughout the tile grid. Navigate to the “Tiles” menu then select “Focus Surface and Support Points” submenu. In the “Multiple Support Points” heading, enter four columns and four rows. Select all wells and then select “distribute”. In the “Interpolation Degree” subheading, choose “2 – Parabolic Saddle Surface (at least 9 support points)”.
8. In the main menu, select “Live”. Navigate to “Verify Support Points” heading and select “Verify”.
9. Adjust the focus throughout the support point. Then, select “Set Z and move to next” until all support points are verified.
10. Under the “Options” subheading, input “10%” for “Tile Overlap”, choose meander for “Travel in Tile Regions”, check “Tile Regions/Positions” box and choose “Sort by Y, then X”, check “Carrier Wells/Container” box and choose “Meander”.
11. Click the “start experiment” button with the tiling parameters in place.
12. Once capture is complete, navigate to the “Processing” tab, and under the “Geometric” subheading, select “Stitching”. Under the “Parameters” subheading, select “Inplace”. Under “Channels” heading, select “All Individually”. Set the parameters as follows for edge detector—”yes”, 5% Minimal Overlap, 10% Max Shift, Optimized for Comparer, and Best for Global Optimizer. Navigate to the “Inputs” submenu and then select the newly generated image from the tile scan.
13. Separate each well into an individual file for easier processing. After the tiles have been stitched, open the “Processing” tab, and under “Utilities”, select “Split Scenes”. Select the stitched image of all of the wells together as “Input” and designate a folder to save the files in; then, select “Apply”.
14. Export the files as.czi.
15. To open.czi in ImageJ, navigate to the Plugins tab and hover over the Bio-Formats option to reveal Bio-Formats Importer. From there, select the file you want to open. A menu will appear asking how these images should be opened. Check the “Use virtual stack” option under the “Memory Management” subheading and select “OK”. A new window will open asking which “Scenes” to open. These are different pixel sizes of the same image. Uncheck scene 1 and scroll down to check the last scene and open the file.
16. Navigate to the Image tab and hover over Type and select 8-bit. To add a scale bar, navigate to Analyze and hover over Tools to select “Scalebar…”. Input the desired size. To save the file, navigate to File, hover over “Save as”, and choose “Jpeg…”.
17. Proceed to image processing in Section 3.7.

Imaging option 2—Bio-Rad ChemiDoc™ MP Imaging System:

1. Place the fixed plates from Section 3.5 right side up without the lid in Bio-Rad ChemiDoc™ MP imaging system using the black background plate.
2. Place a fluorescent metric ruler (commonly used for Bio-Rad calibration) along the edge of the six-well plate.
3. Select Cy5.5 imaging setting and adjust the exposure to between 1 and 2 s of exposure or use the auto-exposure feature.
4. Collect image. Proceed to image processing in Section 3.7.

Imaging option 3—Smartphone camera:

1. Place a white paper on a benchtop. Next, place the fixed plates from Section 3.5 upside-down on the paper. Alternatively, a light box such as those used to image Coomassie-stained gels can be used for better illumination.
2. Place a metric ruler along the edge of the 6-well plate if the plaque area is desired in the calculations.
3. Hold the smartphone parallel to the plate and tap the screen to focus on the well of interest. Hold the phone to avoid shadowing on the well of interest.
4. Collect image. Be sure to convert the photo file to .jpg file type if needed. Proceed to image processing in Section 3.7.

### 3.7 Image processing

Note that processing images will require some optimization of the set to be processed. Once the parameters are optimized, apply the parameters to all wells imaged. This protocol was developed for hCMV plaques, which appear as darker areas of staining on a lighter background of cell monolayer.

**Figure 3:**
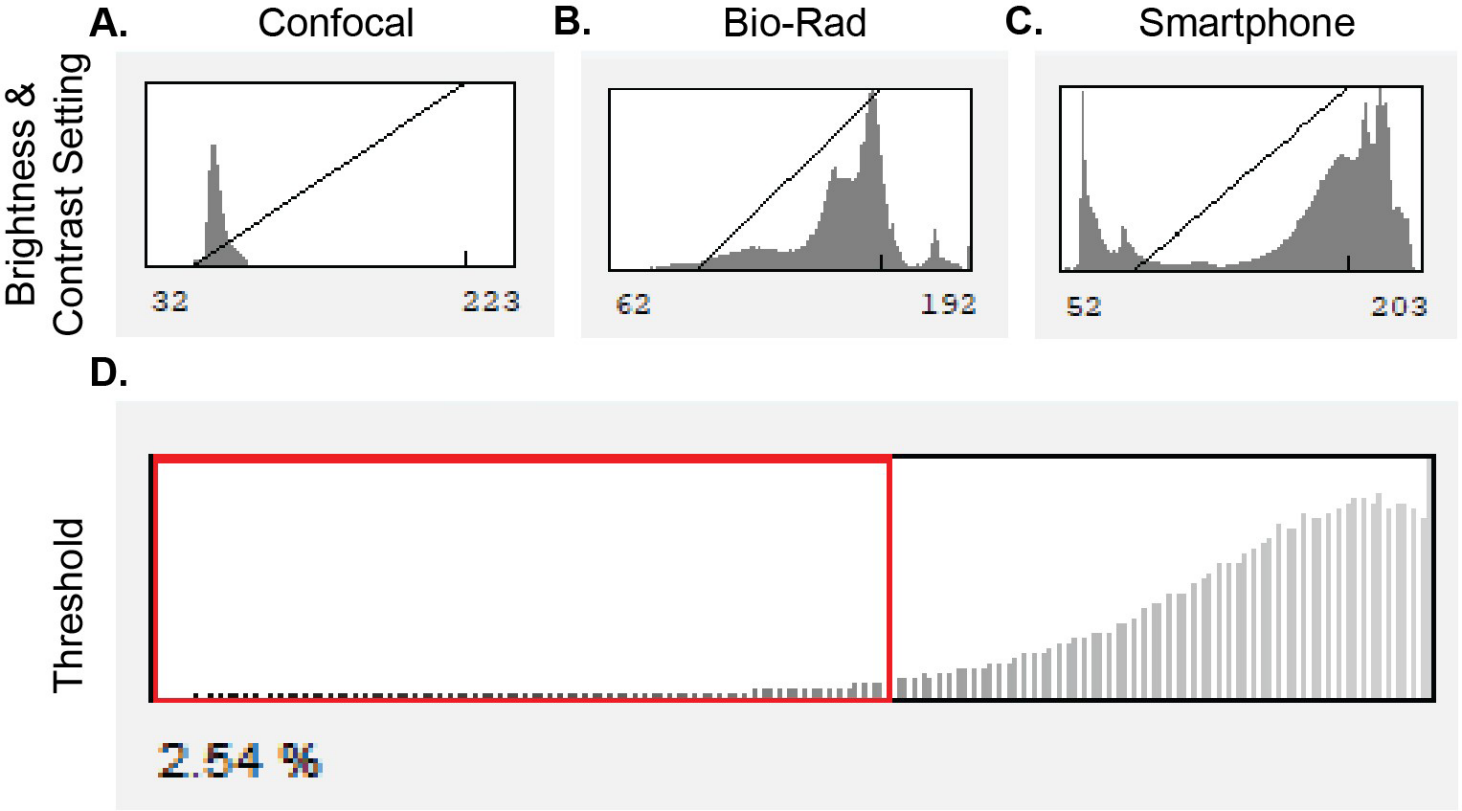
Example histograms indicating how to apply the threshold. **(A)** Example histogram of contrast from confocal imaging with a diagonal line illustrating how to adjust contrast to the base of the curve. **(B)** Example histogram of contrast from Bio-Rad imager with a diagonal line illustrating how to adjust contrast to the peak of the curve. **(C)** Example histogram of contrast from smartphone imaging with a diagonal line illustrating how to adjust contrast to the middle of the bimodal curves. **(D)** Example positioning of threshold level.

1. Open the image file in ImageJ. Ensure that the file is an 8-bit by selecting “Image” tab, then under “Type” menu, click “8-bit.”.
2. For images acquired on Bio-Rad ChemiDoc™ MP imaging system or smartphone, use the line tool to trace 1 cm along the top edge of the ruler. Then, in the “Analyze” tab, select “Set Scale”. The length of the traced line will auto-populate in pixels. Ensure that “Known distance” is set to 1 and “Unit of length” is set to cm. Select “OK” to confirm scale.
3. Outline the well of interest using the circle drawing tool.
4. Adjust the contrast and brightness of the image so that the diagonal contrast line is just touching the edge of the side of peak on confocal images (**Figure 3A**) the top of the singular peak in Bio-Rad Chemdoc™ images (**Figure 3B**), or positioned immediately after the first of the two peaks in the smartphone images (**Figure 3C**). This is done by selecting the “Image” tab and then selecting the “Adjust” tab.
5. Apply a 1.5% Gaussian blur. This is found in the “Process” tab under the “Filters” menu. This step allows ImageJ to smoothen the edges of the plaques and ignore the sharper edges in the plaques.
6. Adjust the threshold to include ~2.5%–5% of pixels (**Figure 3D**). Threshold parameters are set in the “Image” tab and then select the “Adjust” tab. This step allows for the most optimization to collected images. Use the mask available in ImageJ to adjust the threshold values until the mask overlaps with the plaque area as desired. There will be some areas of the threshold highlighted outside of the desired plaques due to some variation in cell uptake of stain. These small areas will be filtered out at a later step, optimizing full plaque coverage.
7. In the “Process” tab, access the “Binary” menu and select “fill holes”.
8. Select the “Analyze” tab and choose “Analyze particles”. Here set the lower size limit as needed to filter out background cell noise that did not meet the threshold requirements.
9. Export data to Excel or other data processing applications. Data include plaque size and counts.
10. Repeat the analysis with all of the collected images using parameters optimized on test image.

## 4 Results

### 4.1 Quantification of hCMV plaques by crystal violet fluorescence using confocal Cy5.5 channel

**Figure 4:**
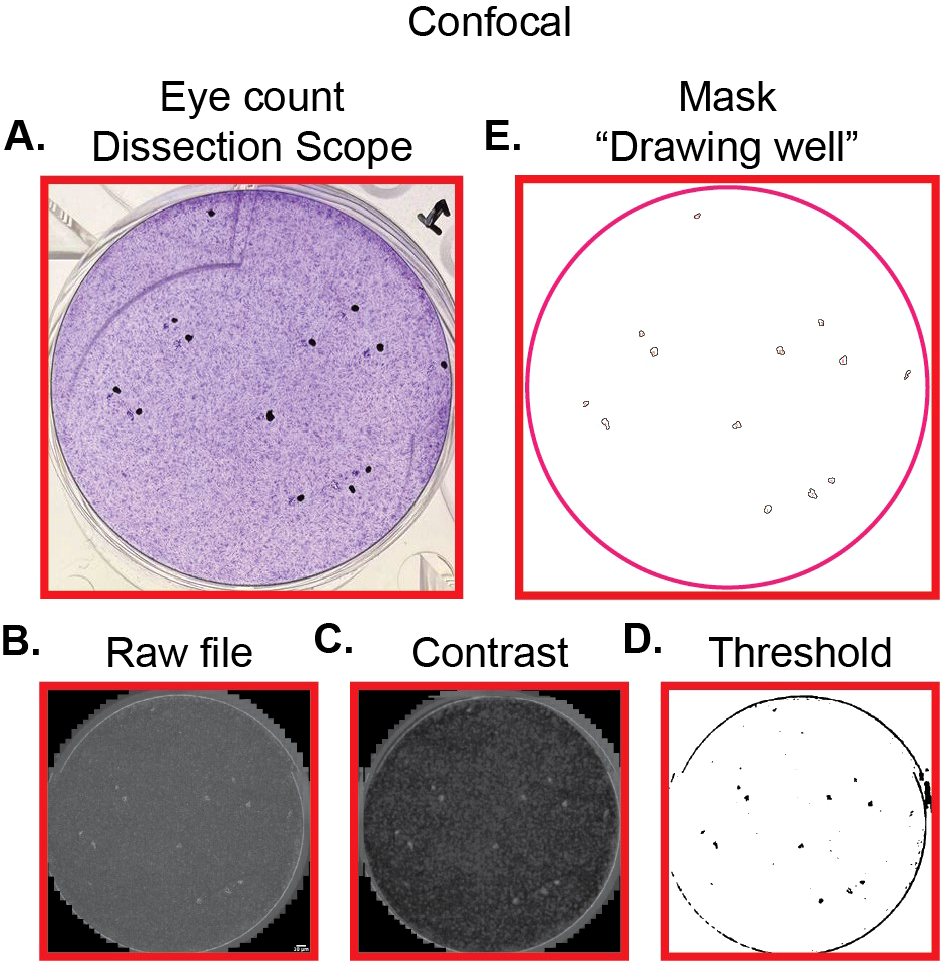
Representative set of images for pipeline quantifying hCMV plaques using confocal microscope Zeiss LSM 980 with Airyscan 2. **(A)** Photograph of hCMV plaques marked in black to highlight regions with plaque formation. Plaques are seen just to the left of the black dots. **(B)** Raw file of hCMV plaques captured as detailed in Section 3.6—option 1 using 647 nm excitation and a confocal microscope. **(C)** Image from **(B)** following contrast adjustment and Gaussian filter as described in Section 3.7 and **Figure 3A**. **(D)** Image from **(C)** following threshold settings as described in Section 3.7 and **Figure 3D**. **(E)** Outline of plaques generated from ImageJ pipeline.

We first hypothesized that based on our previous success in quantifying hCMV plaques using the specialized Molecular Devices ImageXpress Micro high-content imaging system, ImageJ and Crystal Violet fluorescence could be generalized to other confocal microscopy systems. To investigate this hypothesis, we quantified the plaque count of the same wells by both traditional hand-counting and by images acquired on Zeiss LSM 980 with Airyscan 2 and our ImageJ pipeline. This comparison was done in triplicate. We found that our pipeline detected 38 of 40 total plaques identified via hand-counting. Furthermore, no background regions were counted as plaques. This amounts to 94.4% ± 0.5% sensitivity and 100% precision of the test. This suggests that we have developed a robust pipeline to identify hCMV plaques using crystal violet fluorescence. As a function of this counting tool, the area of each plaque is also measured without the need to hand-trace any plaques. An example of one well that was counted and optimized for confocal microscopy by image processing in Section 3.7 is shown in **Figure 4**.

### 4.2 Quantification of hCMV plaques by crystal violet fluorescence using Bio-Rad ChemiDoc™ MP Cy5.5 channel

As the Cy5.5 channel is available in Bio-Rad imaging systems, we next investigated whether these systems can be used for imaging wells stained with crystal violet. We compared six wells imaged by the Bio-Rad imaging system followed by our ImageJ pipeline as above to hand-counted standards.

**Figure 5:**
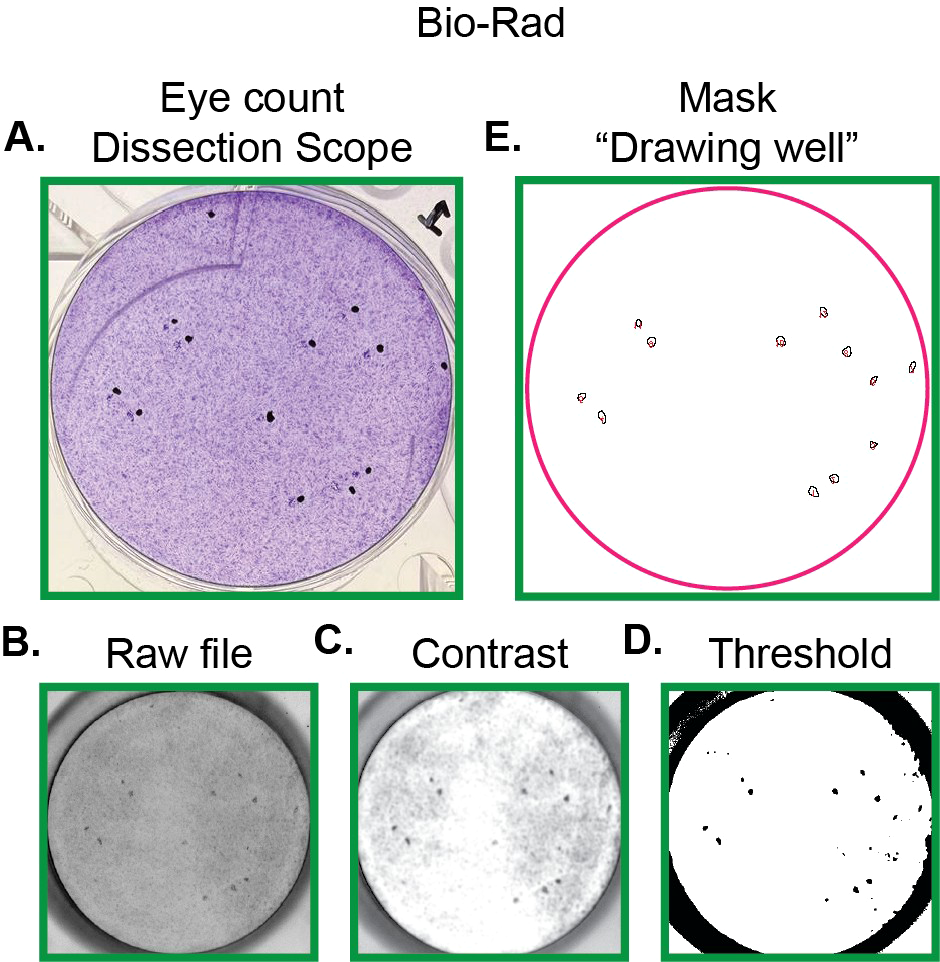
Representative set of images for pipeline quantifying hCMV plaques using Bio-Rad imaging system. **(A)** Photograph of hCMV plaques marked in black to highlight regions with plaque formation. Plaques are seen just to the left of the black dots. For ease of comparison, the same well presented in **Figure 4** is shown here. **(B)** Raw file of hCMV plaques captured as detailed in Section 3.6—option 2. **(C)** Image from **(B)** following contrast adjustment and Gaussian filter as described in Section 3.7 and **Figure 3B**. **(C)** Image from **(C)** following threshold settings as described in Section 3.7 and **Figure 3D**. **(E)** Outline of plaques generated from ImageJ pipeline.

In this trial, the ImageJ pipeline was able to detect 81.4% ( ± 3.1% SE) of plaques in the imaged wells on average. This value represents the specificity of our pipeline for this dataset. Additionally, the regions identified as plaques by hand were located under the dissection microscope in 92.1% ( ± 1.7% SE) of cases. This represents a high precision of identifying plaques. However, this method has reduced sensitivity compared to the use of confocal microscopy. An example of one well that was counted and optimized for the Bio-Rad imaging systems from Section 3.7 is shown in **Figure 5**.

### 4.3 Quantification of hCMV plaques by crystal violet fluorescence using a smartphone camera

Given the near-ubiquitous nature of smartphones with rapidly increasing camera technology, we hypothesized that these smartphone cameras could be used to acquire images for our ImageJ pipeline. To test this, we acquired images from 12 wells. Images were run through pipeline as described in Section 2.3.

**Figure 6:**
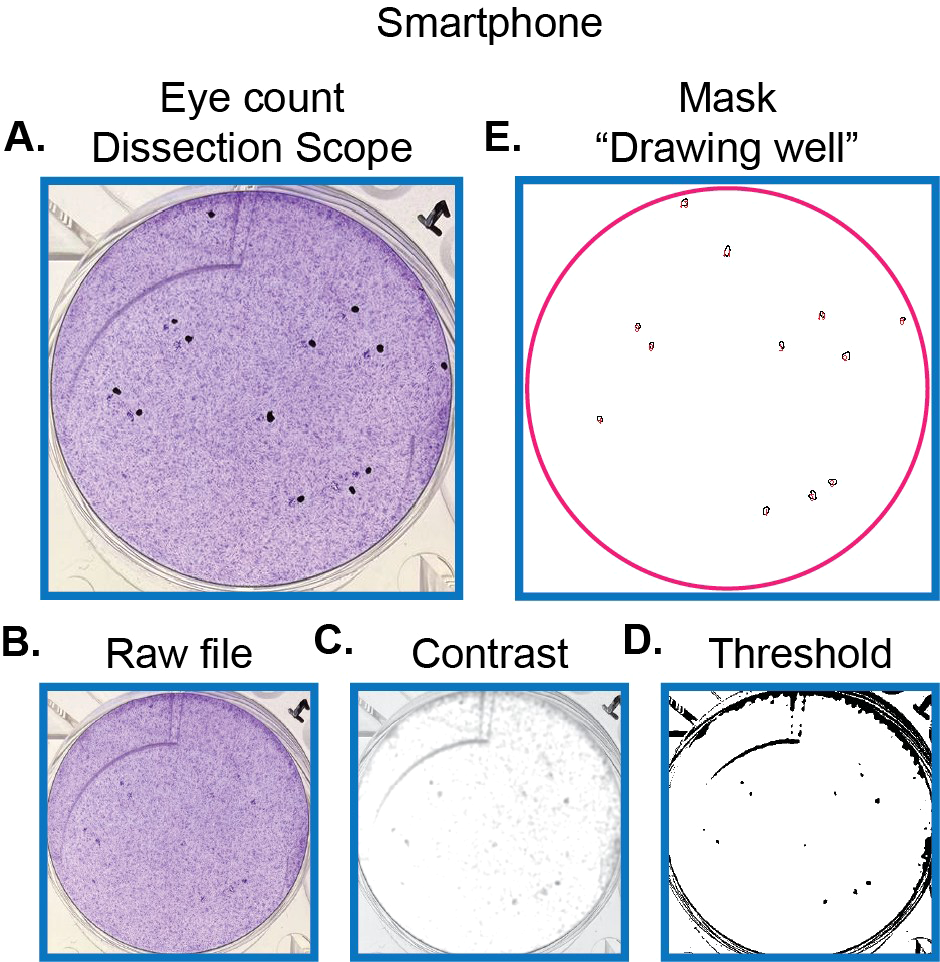
Representative set of images for pipeline quantifying hCMV plaques using a smartphone camera. **(A)** Photograph of hCMV plaques marked in black to highlight regions with plaque formation. Plaques are seen just to the left of the black dots. For ease of comparison, the same well presented in **Figures 4** and **5** is shown here. **(B)** Raw file of hCMV plaques captured as detailed in Section 3.6—option 3. **(C)** Image from **(B)** following contrast adjustment and Gaussian filter as described in Section 3.7 and **Figure 3C**. **(D)** Image from **(C)** following threshold settings as described in Section 3.7 and **Figure 3D**. **(E)** Outline of plaques generated from ImageJ pipeline.

In this trial, the ImageJ pipeline was able to detect 82.5% ( ± 3.4% SE) of plaques in the imaged wells on average. This value represents the specificity of our pipeline for this dataset. Additionally, the regions identified as plaques by hand were located under the dissection microscope in 91.2% ( ± 2.9% SE) of cases. This once again represents a high precision of identifying plaques. An example of one well that was counted and optimized for smartphone cameras by image processing as detailed in Section 3.7 is shown in **Figure 6**.

## 5 Discussion

**Figure 7:**
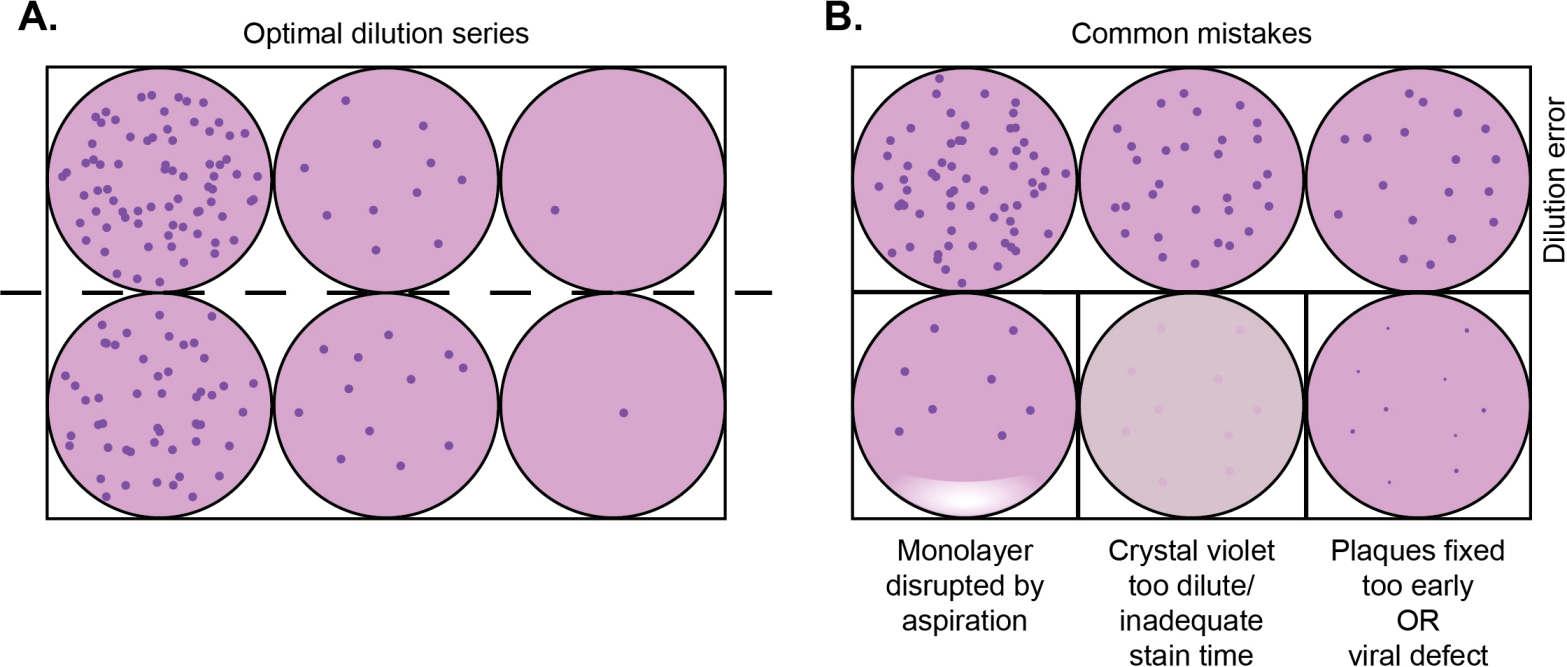
Illustration of optimal plaque setup and common mistakes that can affect reliable imaging and the analysis of plaque assays. **(A)** Schematic of an optimal 10-fold dilution series starting from left to right shown in duplicate for clarity, although we recommend performing in triplicate. **(B)** Top row: Error during the dilution series without 10-fold fewer plaques in subsequent wells. This may be due to the inadequate mixing of viral dilutions, use of the same pipet tip in numerous dilutions, or biologic variation of virus causing clumping. Bottom left: Disruption of the cell monolayer caused by the tip contacting the monolayer or by the aspiration of too much liquid from the well. Bottom middle: Staining with lower crystal violet percentages can cause the plaques to be less visible. Bottom right: The incubation period for the entire plaque assay was too short, as per **Table 1**, or there may have been a biological change in the virus if the control samples have larger plaques.

With this protocol, we successfully streamlined the hCMV plaque assay combined with readily accessible software. We provide instructions on adapting images captured in various modalities. While optimizing our bench protocol to allow for more reliable quantification of these plaques, we have identified several common errors and ways to avoid these pitfalls (**Figure 7**).

As this pipeline relies on the collection of clean images, many of the limitations of this protocol are related to image collection. Most notably, imaging of a large surface area of an entire well can be time intensive and generate large data files. The use of both smart phones and the Bio-Rad ChemiDoc™ MP imaging system is limited by the presence of shadows or distortions caused by the plastic of the wells. Regardless of these limitations, there are also advantages that come with each imaging modality (summarized in **Table 3**).

**Table 3:** Advantages and disadvantages of each imaging modality.

Image type	Advantages	Disadvantages
Confocal microscopes	Best resolution on plaque size Imaging on far red channel leads to improved contrast to distinguish plaques from background	Large file size Time to acquire and stitch images is significantly higher than other options Specialized machinery not widely available
Express plate imager	Efficient and high resolution Can also use fluorescence	Specialized machinery not widely available Some resolution trade-off for speed
Smartphone camera	Widely available and does not require training on specialized machinery Smaller file sizes while still maintaining an accurate representation of the well	Can introduce parallax Shadowing from the plastic well can distort image and cause background
Bio-Rad Imager	Commonly available machine with minimal training required Consistent imaging Can also use fluorescence	Can have significant shadow and background Fluorescent option is not always available

Summary of the advantages and disadvantages of using confocal microscopy, express plate imager, smartphone camera, or Bio-Rad Imager for collecting images and the subsequent analysis as described in sections 3.6-6.7.

We have optimized this pipeline based on the characteristics of the images acquired. Once the parameters of the pipeline were established, the pipeline was accurately applied between several images in the same collection modality. This suggests that pipelines may be readily applied to fixed plaque assays from other species of hCMV or even plaques generated from other viruses. This method may also be generalizable to other dyes for staining. Methylene blue and neutral red dyes have both been used in plaque assays, and both are weakly fluorescent (Kirk, 1970; Schmidt et al., 1976; Ghiringhelli and Romanowski, 1994; Cwalinski et al., 2020).

## 6 Recipes

### 6.1 2% methylcellulose w/v

Note: 2% methylcellulose is highly viscous. Aliquot by ensuring that the lip of the bottle is clean and pour into aliquot bottles in a biosafety cabinet rather than attempting to pipet.

1. Weigh 20 g of methylcellulose and place in a 1-L glass bottle (**Table 4**). Place a magnetic stir bar in with methylcellulose. Lightly place cap on the bottle. IMPORTANT: Do not tighten the cap.
2. Measure 1 L of ultrapure water into a separate 1-L Corning bottle. Lightly place the cap on the bottle. IMPORTANT: Do not tighten the cap.
3. Autoclave both bottles using a 15-min minimum wet/liquid cycle. IMPORTANT: Autoclaved materials will be hot. Use appropriate PPE when handling. Allow the water to cool until the bottle can be comfortably handled. It is also critical to proceed while the materials are still quite warm. If the water cools too much, the methylcellulose will not fully dissolve.
4. Inside a TC hood, slowly pour the autoclaved water into the glass bottle containing methylcellulose.
5. Stir the solution at room temp for 10 min.
6. Let the solution continue to stir overnight at 4°C.
7. Aliquot into 500-mL bottles in a TC hood. Store at 4°C.

**Table 4:** 2% methylcellulose w/v recipe Recipe for 1L of 2% methylcellulose w/v used in section 3.4.

Reagent	Amount needed	Final concentration
Methylcellulose	20 g	2%
Ultrapure water	1 L	

For catalog numbers of products used in this recipe see section 2.1.

### 6.2 1% crystal violet w/v

Note: Crystal violet will stain the skin, clothing, and some types of plastic. Wear appropriate personal protective equipment (PPE) including lab coat and gloves when handling. Corning bottles should be dedicated to crystal violet as it will also stain glassware and may interfere with other reagents mixed in the same glassware. Consult with institution disposal and storage guidelines prior to mixing.

1. Weigh 10 g of anhydrous crystal violet and add to a 1-L glass bottle (**Table 5**).
2. Add 800 mL of ultrapure water and a magnetic stir bar to the glass bottle.
3. Stir at room temperature until the crystal violet is dissolved.
4. Add 200 mL of methanol to the bottle and continue stirring until the solution has been thoroughly mixed.

**Table 5:** 1% crystal violet w/v recipe Recipe for 1L of 1% crystal violet w/v used in section 3.5.

Reagent	Amount needed	Final concentration
Crystal violet	10 g	1%
Methanol	200 mL	20%
Ultrapure water	800 mL	

For catalog numbers of products used in this recipe see section 2.1.

### 6.3 Serum-free DMEM

1. In a biosafety cabinet, add 5 mL of penicillin–streptomycin to 500 mL of high-glucose DMEM (**Table 6**).
2. Store at 4°C.
3. Heat to 37°C in a water bath prior to use.

**Table 6:** Serum-free DMEM Recipe for 500 mL of Serum-free DMEM used in section 3.

Reagent	Amount needed
High-glucose DMEM	500 mL
Penicillin–streptomycin 10,000 U/mL	5 mL

For catalog numbers of products used in this recipe see section 2.1.

### 6.4 DMEM with 4% FBS

Note: For exact serum concentration, remove 25 mL from the bottle of DMEM and replace with fetal bovine serum (FBS) and penicillin–streptomycin.

1. In a biosafety cabinet, add 5 mL of penicillin–streptomycin and 20 mL of heat-inactivated FBS to 500 mL of high glucose DMEM (**Table 7**).
2. Store at 4°C.
3. Heat to 37°C in a water bath prior to use.

**Table 7:** DMEM with 4% FBS Recipe for 500 mL of DMEM with 4% FBS used in section 3.

Reagent	Amount needed
High-glucose DMEM	500 mL
Penicillin–streptomycin 10,000 U/mL	5 mL
FBS	20 mL

For catalog numbers of products used in this recipe see section 2.1.

### 6.5 DMEM with 10% FBS

Note: For exact serum concentration, remove 55 mL from the bottle of DMEM and replace with FBS and penicillin–streptomycin.

1. In a biosafety cabinet, add 5 mL of penicillin–streptomycin and 50 mL of heat-inactivated FBS to 500 mL of high-glucose DMEM (**Table 8**).
2. Store at 4°C.
3. Heat to 37°C in a water bath prior to use.

**Table 8:** DMEM with 10% FBS Recipe for 500 mL of DMEM with 10% FBS used in section 3.

Reagent	Amount needed
High-glucose DMEM	500 mL
Penicillin–streptomycin 10,000 U/mL	5 mL
FBS	50 mL

For catalog numbers of products used in this recipe see section 2.1.

## Data Availability

The datasets presented in this study can be found in online repositories. The names of the repository/repositories and accession number(s) can be found in the article/**Supplementary Material**.
